# Functional Significance of GnRH and Kisspeptin, and Their Cognate Receptors in Teleost Reproduction

**DOI:** 10.3389/fendo.2013.00024

**Published:** 2013-03-08

**Authors:** Renjitha Gopurappilly, Satoshi Ogawa, Ishwar S. Parhar

**Affiliations:** ^1^Brain Research Institute, School of Medicine and Health Sciences, Monash University Sunway CampusSelangor, Malaysia

**Keywords:** GnRH receptor, kisspeptin receptor, GPR54, reproduction, teleost fish

## Abstract

Guanine nucleotide binding protein (G-protein)-coupled receptors (GPCRs) are eukaryotic transmembrane proteins found in all living organisms. Their versatility and roles in several physiological processes make them the single largest family of drug targets. Comparative genomic studies using various model organisms have provided useful information about target receptors. The similarity of the genetic makeup of teleosts to that of humans and other vertebrates aligns with the study of GPCRs. Gonadotropin-releasing hormone (GnRH) represents a critical step in the reproductive process through its cognate GnRH receptors (GnRHRs). Kisspeptin (Kiss1) and its cognate GPCR, GPR54 (=kisspeptin receptor, Kiss-R), have recently been identified as a critical signaling system in the control of reproduction. The Kiss1/Kiss-R system regulates GnRH release, which is vital to pubertal development and vertebrate reproduction. This review highlights the physiological role of kisspeptin-Kiss-R signaling in the reproductive neuroendocrine axis in teleosts through the modulation of GnRH release. Moreover, we also review the recent developments in GnRHR and Kiss-R with respect to their structural variants, signaling mechanisms, ligand interactions, and functional significance. Finally, we discuss the recent progress in identifying many teleost GnRH-GnRHR and kisspeptin-Kiss-R systems and consider their physiological significance in the control of reproduction.

## Introduction

Guanine nucleotide binding protein (G-protein)-coupled receptors (GPCRs) play a pivotal role in various physiological processes (Marinissen and Gutkind, 2001), and GPCRs are now recognized as major pharmaceutical drug targets (Shoichet and Kobilka, 2012). GPCR sequencing in model organisms has proven vital for identifying novel GPCRs and their ligands with potential therapeutic value (Metpally and Sowdhamini, 2005). Evolutionary comparisons of GPCR sequences may allow for the identification of conserved motifs and the recognition of key functional residues (Attwood, 2001; Bjarnadottir et al., 2005). As research models, fish have been used largely to exploit their aquaculture potential. However, in recent years, there has been a trend toward using them in biomedical research as models of human disease. Because fish are phylogenetically diverse, they can be used to understand the fundamental principles of vertebrate evolution and disease processes. Therefore, the teleost genome provides an additional model to study the evolution and function of GPCRs (Metpally and Sowdhamini, 2005).

The hypothalamic-pituitary-gonadal (HPG) axis regulates puberty in vertebrates, primarily through the hypothalamic secretion of gonadotropin-releasing hormone (GnRH). This decapeptide hormone stimulates the release of the following gonadotropins from the anterior pituitary: follicle-stimulating hormone (FSH) for gamete growth and luteinizing hormone (LH) for gamete maturation and release (Ankley and Johnson, 2004; Weltzien et al., 2004). Recently, kisspeptin, a novel neuropeptide encoded by the metastasis suppressor gene, Kiss1/KISS1 (rodents/human), and its cognate GPCR, kisspeptin receptor (Kiss-R) (=GPR54), have been identified as potent regulators of reproduction, particularly for the onset of puberty (de Roux et al., 2003; Seminara et al., 2003). The kisspeptin-GPR54 system is vital to the onset of puberty, as it is critical to the activation of GnRH neurons. In 2004, we were the first to report the expression of GPR54 mRNA in GnRH neurons in a cichlid fish, tilapia, which strongly supports this concept (Parhar et al., 2004). Following this discovery, the presence of GPR54 in GnRH neurons has been demonstrated in several mammalian and non-mammalian species (Irwig et al., 2004; Han et al., 2005; Messager et al., 2005; Grone et al., 2010). Moreover, the innervation of kisspeptin fibers to GnRH neurons has been illustrated in several vertebrates (Clarkson and Herbison, 2006; Ramaswamy et al., 2008; Servili et al., 2011). The HPG axis is regulated by a number of GPCRs that play important roles in reproduction and sex hormone-dependent diseases (Heitman and Ijzerman, 2008). These receptors are therefore referred to as “reproductive” GPCRs. Numerous GPCRs with roles in reproduction have been discovered in recent years (Millar and Newton, 2010). In this review, we focus on the GnRH receptor (GnRHR), the recently deorphanized Kiss-R, and the regulation of GnRH secretion by an intricate interplay between the two in the teleosts.

## GnRH Receptor Types

### Structural variants

Gonadotropin-releasing hormone is the hypothalamic decapeptide, which is mainly responsible for reproductive function. It is secreted from the hypothalamus into the hypophyseal portal circulation via the median eminence, which binds and activates the GnRHR that is expressed on the surface of the pituitary gonadotrope cells in mammals (Neill, 2002). In teleosts, the equivalent of the median eminence is incorporated into the rostral neurohypophysis of the pituitary (Batten and Ingleton, 1987). GnRH fibers have been observed to directly innervate the neurohypophysis in the pituitary (Kah et al., 1986; Parhar et al., 2002). In the pituitary, GnRH interacts with high-affinity GnRHRs in cell membranes of gonadotrophs, leading to biosynthesis and secretion of LH and FSH (Karges et al., 2003). To date, more than 20 forms of GnRH and the corresponding genes have been identified (Tsai, 2006; Chen and Fernald, 2008; Okubo and Nagahama, 2008; Roch et al., 2011; Tostivint, 2011; Kochman, 2012). These have a broad range of functions, including neuroendocrine, neurotransmitter/neuromodulatory, paracrine and autocrine functions (Millar et al., 2004), and each GnRH type is capable of serving all these roles. Structural and phylogenetic analysis of the GnRH genes classifies GnRH types into three forms (White and Fernald, 1998). Most vertebrate species possess at least two or three GnRH forms (Sherwood, 1987; Sherwood et al., 1993; Sealfon et al., 1997; Millar, 2003). GnRH-II (=GnRH2) is ubiquitously found in the most vertebrates, which was first isolated from the chicken (cGnRH-II) (Miyamoto et al., 1984). Because GnRH2 structure is highly conserved in the vertebrate species, it most likely serves critical functions and evolved the earliest (Millar et al., 2004). The hypothalamic form is designated GnRH type I (GnRH1) (Troskie et al., 1998), which is the species-specific form and regulates pituitary LH release (Parhar, 2002), and most of which have been identified in fish (Okubo and Nagahama, 2008). A third GnRH type (salmon GnRH-III = GnRH3) is only exhibited in the forebrain of teleost fish. A recent gene synteny analysis of the genomic regions comprising fish GnRH3 has found similar arrangement of GnRH3 gene cluster in the tetrapod genomes (Roch et al., 2011), which indicates that tetrapod GnRH3 was lost after divergence of fish and tetrapod lineages (Kim et al., 2011; Tostivint, 2011). Lamprey GnRH-I and -III, have recently been categorized as GnRH type IV (GnRH4), exclusive to the jawless fish (Roch et al., 2011).

GnRH receptor gene sequence was first identified from the mouse αT3 gonadotrope cell line (Tsutsumi et al., 1992; Millar et al., 2004). The ligand binding pocket of the GnRHR is formed by residues in the extracellular loops and the interior of transmembrane helices, indicating that GnRH partially enters the transmembrane core (Forfar and Lu, 2011). Mammalian GnRHR homologs have been isolated from non-mammalian species such as reptile, amphibians, birds, teleosts, and invertebrate species (Millar, 2003). The first teleost GnRHR was isolated from the African catfish (Tensen et al., 1997), and piscine GnRHR homologous sequences have been isolated from various teleosts such as the goldfish (Illing et al., 1999), Japanese eel (Okubo et al., 2000), trout (Madigou et al., 2000), striped bass (Alok et al., 2000), cichlid (Robison et al., 2001; Parhar et al., 2005a), medaka (Okubo et al., 2001), salmon (Jodo et al., 2003), fugu (Moncaut et al., 2005), and cobia (Mohamed et al., 2007). In some teleosts, there are four or five GnRHR isoforms (Jodo et al., 2003; Moncaut et al., 2005). Recent phylogenetic analyses based on amino acid sequence identity have classified teleosts GnRHR isoforms into three major groups (Mollusk et al., 2011; Roch et al., 2011). Furthermore, a recent classification based on genome synteny analysis has also revealed three major lineages of fish GnRHR types that further subdivide into five classes: non-mammalian type I (GnRHRn1 and GnRHRn1b), non-mammalian type II (GnRHRn2), and non-mammalian type III (GnRHRn3 and GnRHRn3b) (Kim et al., 2011) (Table 1). The genome synteny-based classification has clearly demonstrated high conservation of neighboring genes of GnRHR types, which are also found in tetrapod GnRHR containing genome fragments (Kim et al., 2011). The neighboring gene sets of fish GnRHRn1, found in GnRHRn1b are probably generated by the teleost-specific third round genome duplication (Kim et al., 2011).

**Table 1:** **GnRH receptors in teleosts**.

Type	Species	Original name	Accession number/ensembl ID	Ligand selectivity	Localization		Reference
					Brain	Pituitary	
GnRHRn1	Medaka	GnRHR3	NP_001098393	GnRH2 = GnRH3 > GnRH1	+	+	Okubo et al. (2003)
	Zebrafish	GnRHR4	NP_001091663	ND	ND	ND	
	Green pufferfish	GnRHR1/III-3	BAE45698	ND	ND	ND	
	Fugu	GnRHR-II-like	XP_003967097	ND	ND	ND	
	Stickleback	GnRHR	ENSGACP00000014249	ND	ND	ND	
	European seabass	GnRHR2B	CAE54807	ND	+	−	Moncaut et al. (2005)
	Platyfish	GnRHR	ENSXMAP00000002818	ND	ND	ND	
	Cod	GnRHR	ENSGMOP00000013942	ND	ND	ND	
	Tilapia	GnRHR-II-like	XP_003440455	ND	+	+	Soga et al. (2005)
	Coelacanth	GnRHR	ENSLACP00000020711	ND	ND	ND	
	*Astatotilapia*	GnRHR1	AAK29745	GnRH2 > GnRH3 > GnRH1	+	+	Robison et al. (2001)
GnRHRn1b	Medaka	GnRHR1	NP_001098352	GnRH2 ≥ GnRH3 = GnRH1	ND	ND	Okubo et al. (2001)
	Green pufferfish	GnRHR1/III-1	BAE45694	ND	ND	ND	
	Fugu	GnRHR	ENSTRUP00000018665	ND	ND	ND	
	Stickleback	GnRHR	ENSGACP00000019583	ND	ND	ND	
	European seabass	GnRHR2C	CAE54805	ND	+	+	Moncaut et al. (2005)
	Platyfish	GnRHR	ENSXMAP00000011787	ND	ND	ND	
GnRHRn2	Zebrafish	GnRH-R2	NP_001138451	ND	ND	ND	
	Medaka	GnRHR	ENSORLP00000015859	ND	ND	ND	
	Green pufferfish	GnRHR1/III-2	BAE45696	ND	ND	ND	
	Fugu	GnRHR	ENSTRUP00000014430	ND	ND	ND	
	Stickleback	GnRHR	ENSGACP00000021774	ND	ND	ND	
	European seabass	GnRHR	CAD11992	ND	+	+	Madigou et al. (2000)
	Cod	GnRHR	ENSGMOP00000000888	ND	ND	ND	
	Platyfish	GnRHR	ENSXMAP00000018201	ND	ND	ND	
GnRHRn3	Zebrafish	GnRHR3	NP_001170921	ND	ND	ND	
	Medaka	GnRH-R2	NP_001098392	GnRH2 > GnRH3 > GnRH1	ND	ND	Okubo et al. (2001)
	Green pufferfish	GnRH-R2/nmI-2	BAE45702	ND	ND	ND	
	Fugu	GnRHR	ENSTRUP00000014399	ND	ND	ND	
	Stickleback	GnRHR	ENSGACP00000004101	ND	ND	ND	
	European seabass	GnRHRII	AAS49921	ND	+	+	Moncaut et al. (2005)
	Coelacanth	GnRHR	ENSLACP00000018841	ND	ND	ND	
	Tilapia	GnRHR	ENSONIP00000001826	ND	ND	ND	
	*Astatotilapia*	GnRH-R2	AAU89433	ND	+	+	Chen and Fernald (2006)
GnRHRn3b	Zebrafish	GnRHR1	NP_001138452	ND	ND	ND	
	Green pufferfish	GnRH-R2/nmI-1	BAE45702	ND	ND	ND	
	*Fugu*	GnRHR	ENSTRUP00000014399	ND	ND	ND	
	Stickleback	GnRHR	ENSGACP00000000651	ND	ND	ND	
	European seabass	GnRHR1A	CAE54804	ND	+	+	Moncaut et al. (2005)
	Cod	GnRH-R1b	ADD92008	ND	ND	ND	
	Goldfish	GnRHRA	AAD20001	GnRH2 > GnRH3 > mGnRH1	+	+	Illing et al. (1999)
	Goldfish	GnRHRB	AAD20002	GnRH2 > GnRH3 > mGnRH1	+	+	Illing et al. (1999)

*ND, not determined*.

### Localization and function of GnRHR types

Localization of the various GnRHRs may provide some insight into their role in the physiology of reproduction. Five teleost GnRHR types (GnRHRn1, GnRHRn1b, GnRHRn2, GnRHRn3, and GnRHRn3b) have been localized in various reproductive organs including the gonads, brain, and pituitary (Lethimonier et al., 2004). In the European sea bass, five GnRHR genes show differential expression in various tissues, in which GnRHR1A (GnRHRn3b) and GnRHR (GnRHRn2) are widely distributed in reproductive and non-reproductive tissues including the eye, kidney, gills, gut, and liver, whereas GnRHRII (GnRHRn3), GnRHR2B (GnRHRn1), and GnRHR2C (GnRHRn1b) are more restricted to the central nervous system (Moncaut et al., 2005). This finding suggests multiple neuroendocrine and neuromodulatory roles of GnRH types throughout the body of teleosts (Jodo et al., 2003).

In the brain, the distribution of GnRHR types has been studied by RT-PCR, *in situ* hybridization and immunohistochemical approaches (Table 1). *In situ* hybridization studies in the European seabass have demonstrated the expression of GnRHR (GnRHRn2) in the forebrain, and the midbrain (Gonzalez-Martinez et al., 2004). In *Astatotilapia burtoni*, GnRHR1 (classified as GnRHRn1 based on sequence homology) is expressed in restricted brain regions including the telencephalon, preoptic area, ventral hypothalamus, and thalamus, whereas GnRH-R2 (GnRHRn3) is expressed in many more brain areas (Chen and Fernald, 2006). In the tilapia, GnRHR1 (GenBank accession number: XM_003437572) and/or GnRHR3 (XM_003437677) (both are classified as GnRHRn1) immunoreactive cells have been shown in the forebrain and midbrain (Soga et al., 2005). Most GnRHR types are found in brain areas involved in reproductive functions (Volkoff and Peter, 1999), and several GnRHR types are also found in the brain region that is known to be involved in appetite control, feeding, and stress responses (Chandroo et al., 2004; Volkoff et al., 2005).

**Figure 1:**
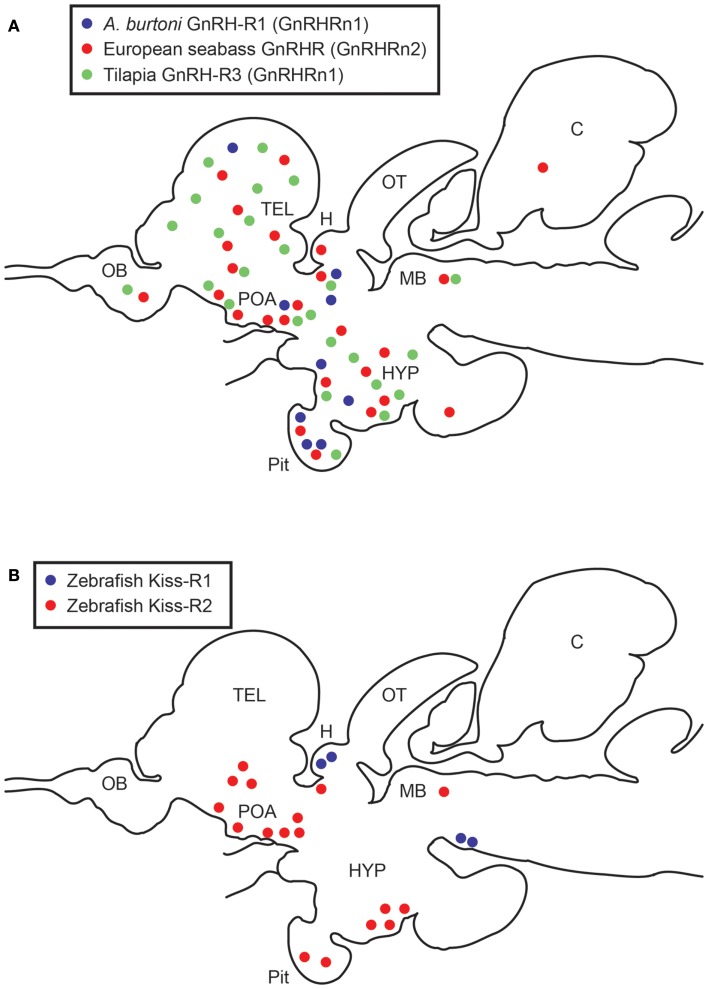
**Schematic diagrams showing distribution of GnRHRs (A) and Kiss-Rs (B)-expressing cells in the brain and pituitary of teleosts**. **(A)** Expression patterns of three GnRHR types expressing cells in three cichlid species, European seabass, *Astatotilapia burtoni*, and Nile tilapia (Gonzalez-Martinez et al., 2004; Soga et al., 2005; Chen and Fernald, 2006). *A. burtoni* GnRHR1 (GnRHRn1), blue; European seabass GnRHR (GnRHRn2), red; Tilapia GnRHR3 (GnRHRn1), green circles. **(B)** Expression patterns of two Kiss-R types expressing cells in the zebrafish brain (Servili et al., 2011; Ogawa et al., 2012a). Kiss-R1 (GPR54b), blue; Kiss-R2 (GPR54a), red circles. OB, olfactory bulb; TEL, telencephalon; POA, preoptic area; H, habenula; OT, optic tectum; HYP, hypothalamus; C, cerebellum; pit, pituitary; MB, midbrain.

*In situ* hybridization studies have demonstrated the expression offish GnRHR in the pituitary: GnRHRn1 [*A. burtoni* GnRHR1, striped seabass GnRHR (AF218841)], GnRHRn1b (European seabass GnRHR2C), GnRHRn2 (European seabass GnRHR, African catfish GnRHR1), GnRHRn3 (*A. burtoni* GnRH-R2, European seabass GnRHRII), and GnRHRn3b [European seabass GnRHR1A, Rainbow Trout GnRHR (NP_001117823), African catfish GnRHR1 (X97497), Goldfish GnRHRA, and Goldfish GnRHRB] (Illing et al., 1999; Alok et al., 2000; Madigou et al., 2000; Gonzalez-Martinez et al., 2004; Moncaut et al., 2005; Chen and Fernald, 2006) (Figure 1A). Within the pituitary cells, most GnRHR types have mainly been localized in the proximal pars distal is of the pituitary where the gonadotrophs (LH and FSH) are exist, which includes GnRHRn1: Tilapia GnRHR1/R3, *A. burtoni* GnRHR1; GnRHRn2: European seabass GnRHR; GnRHRn3/3b: *A. burtoni* GnRH-R2, Rainbow trout GnRHR, Goldfish GnRHRA and GnRHRB (Madigou et al., 2000; Parhar et al., 2002; Gonzalez-Martinez et al., 2004). In some teleosts, the presence of multiple GnRHR types have also been demonstrated in other pituitary cell types such as lactotropes, somatotropes, thyrotropes, melanotropes, corticotropes, and somatolactin cells (Illing et al., 1999; Parhar et al., 2002).

### GnRHR signaling, cycling, and desensitization

**Figure 2:**
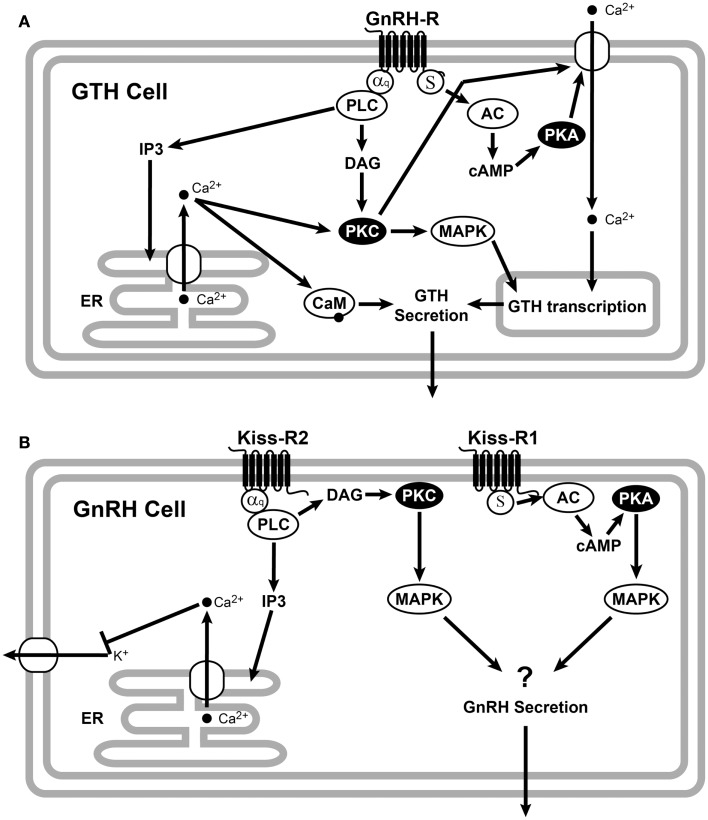
**Schematic representation of GnRHR (A) and Kiss-R (B) signaling**. **(A)** GnRH binds to the Gq/11-coupled membrane receptors. Activation of Gq/11 proteins stimulates phospholipase C (PLC) activity to generate inositol triphosphate (IP3) and diacylglycerol (DAG). Increases of these signal messengers lead to the activation of protein kinase C (PKC) and an increase in intracellular Ca^2+^ concentration from the endoplasmic reticulum (ER). The PKC and Ca^2+^ pathways are involved in the GnRH regulation of GTH subunit gene expression, while GTH secretion is mainly mediated by the GnRH-induced increase in intracellular calcium through the calmodulin (CaM). It has also been proposed that GnRH caused GTP loading on Gs and increased intracellular cAMP via activation of adenylyl cyclase (AC), which elevates GnRH release via the cAMP-dependent protein kinase (PKA) (Liu et al., 2002). **(B)** Binding of kisspeptin to teleosts Kiss-R2 activates the Gq/11 and PLC to generate IP3 and DAG. IP3 causes intracellular Ca^2+^ release from the ER, which activates PKC and mitogen-activated protein kinase (MAPK) cascade. However, teleosts Kiss-R could be activated by the cAMP/PKA pathway probably through Gs. All the figures were adopted from Ando et al. (2001), Krsmanovic et al. (2003), Oakley et al. (2009), d’Anglemont de Tassigny and Colledge (2010), Rønnekleiv et al. (2010).

A recent review by Levavi-Sivan and Avitan (2005) elegantly describes the GnRHR signaling pathway in gonadotrophs (Figure 2A). Mammalian and non-mammalian GnRHRs promote a conformational change in the GnRH peptide structure, which is essential for G-protein coupling and signal transduction (Illing et al., 1999; Cheung and Hearn, 2002; Levavi-Sivan and Avitan, 2005), suggesting similar receptor activation mechanisms. Ligand binding to GnRHR activates phospholipase C to generate inositol (Levavi-Sivan and Avitan, 2005), which mobilizes intracellular Ca^2+^ stores and activates protein kinase C. In teleosts, the cAMP signaling pathway is involved in GnRH release (Yaron et al., 2003; Levavi-Sivan and Avitan, 2005). In the tilapia, GnRH regulates glycoprotein hormone α and LHβ transcription through the PKC and PKA pathways and regulates FSHβ transcription through the PKC and PAPK-independent pathways (Gur et al., 2002). GnRHRs and GPCRs typically undergo desensitization and internalization including receptor phosphorylation, after being activated (McArdle et al., 2002). This phosphorylation stabilizes the association of GPCRs with β-arrestin, which prevents effector activation and acts as an adapter, targeting desensitized GPCRs for internalization (Ferguson, 2001) by endocytosis. Different GnRHR type may have different rates of desensitization and internalization and may also have different repertoire of signaling possibilities (McArdle et al., 2002).

### Regulation of fish GnRHR

GnRH receptor is known to be regulated by several factors. In the tilapia, GnRHR3 has been shown to be up-regulated by its own ligand in the pituitary (Levavi-Sivan et al., 2004). A dopamine-agonist suppressed tilapia GnRHR3 mRNA levels, which indicates an inhibitory effect of dopamine on GnRHR3 synthesis (Levavi-Sivan et al., 2004). Furthermore, tilapia GnRHR3 mRNA levels are higher in vitellogenic females than in maturing males, which could be due to the effect of higher estradiol-17β levels in females on tilapia GnRHR3 mRNA levels (Levavi-Sivan et al., 2004). In female *A. burtoni*, GnRH-R2 mRNA levels are elevated in mouthbrooding female brain compared to fed condition, which could be due to low androgens and estrogens levels (Grone et al., 2012). In rare minnow *Gobiocypris rarus*, bisphenol A, an endocrine disrupting chemical has been recently reported to affect gene expression of GnRHR1A gene in the brains of females (Qin et al., 2013). Although not many studies on promoter analysis of a fish GnRHR have been done, fish GnRHR gene seems to be sensitive to sex steroids as reported in mammals (Hapgood et al., 2005). In the Atlantic cod, pituitary GnRH-R2a but not GnRH-R1b gene expression increases in late-vitellogenic and running females. Continuous light inhibits the increase of pituitary GnRH-R2a expression seen during the normal spawning period (Hildahl et al., 2013). These results suggest that GnRHR is also influenced by nutrition and environmental factors most probably via steroid hormone levels.

## GPR54

Kisspeptin, encoded by the KISS1/Kiss1 gene, is an endogenous ligand for GPR54 (thus called Kiss-R) and promotes GnRH secretion (Kotani et al., 2001; Kaiser and Kuohung, 2005; Dungan et al., 2006). Kiss-R is widely expressed in many reproductive tissues. Mounting evidence suggests the critical role of kisspeptin in the modulation of GnRH secretion in the central nervous system (Irwig et al., 2004; Han et al., 2005; Messager et al., 2005). The kisspeptin-Kiss-R signaling system in various vertebrate species has been described in terms of distribution and physiology (Oakley et al., 2009). This signaling pathway is significant in the reproductive axis in vertebrates, although the location, developmental timing, and expression patterns differ. The anatomy and physiology of piscine kisspeptin-Kiss-R signaling has been extensively reviewed (Elizur, 2009; Zohar et al., 2010; Ogawa and Parhar, 2013). Current knowledge on the gene expression, distribution, and physiological function of piscine kisspeptin and Kiss-R is as follows.

### Kisspeptin receptor types in teleosts

Kisspeptin receptor was first identified as an orphan GPCR (Lee et al., 1999). Mammalian Kiss-R is weakly homologous to galanin receptors (44–45%) but does not bind to either galanin or galanin-like peptides (Lee et al., 1999). We have previously reported a non-mammalian GPR54 for the first time in the tilapia (Parhar et al., 2004). Since then, many groups have identified Kiss-R in several non-mammalian vertebrates. In fish, there are two Kiss-R types (Kiss1Ra/GPR54-1 and Kiss1Rb/GPR54-2 in zebrafish, GPR54a and GPR54b in goldfish) (Lee et al., 2009; Li et al., 2009), while *Xenopus* have three Kiss-R types (GPR54-1a, GPR54-1b, and GPR54-2) (Lee et al., 2009). Genome and cDNA analyses has revealed that the Kiss-R genes contain five exons, although medaka Kiss-R1 and sea bass Kiss-R2 have six exons (Tena-Sempere et al., 2012). Our recent *in silico* study has identified four Kiss-R homologous sequences in an early sarcopterygian, Coelacanth *Latimeria chalumnae* genome, which leads to further clarification of molecular evolution of Kiss-R in vertebrates (describe in below).

Two Kiss-R types have been identified in the Senegalese sole (Mechaly et al., 2009), goldfish (Li et al., 2009), zebrafish (Biran et al., 2008), medaka (Lee et al., 2009), striped bass (Zmora et al., 2012), and sea bass (Tena-Sempere et al., 2012). In Perciform teleosts, the Southern Bluefin Tuna, and the Yellowtail Kingfish, two mRNA transcript variants of Kiss-R2 have been identified (Nocillado et al., 2012). With two different Kiss-R genes, two kisspeptin types (Kiss1 and Kiss2) have been identified in several teleosts such as zebrafish, medaka (Kanda et al., 2008; van Aerle et al., 2008; Kitahashi et al., 2009), goldfish (Li et al., 2009), sea bass (Felip et al., 2009), chub mackerel (Selvaraj et al., 2010), and striped bass (Zmora et al., 2012). Two kisspeptin types have also been identified in *Xenopus* and elephant shark (Lee et al., 2009), which suggests that the kisspeptin-Kiss-R systems function independently, especially in controlling teleost reproduction. Some teleosts possess only one kisspeptin type (Kiss2) or/and its receptor (Kiss-R2) (Tena-Sempere et al., 2012). Previous genome synteny analyses (Um et al., 2010; Tena-Sempere et al., 2012) have suggested that the Kiss-R genes previously identified from tilapia (Parhar et al., 2004), a cichlid *A. burtoni* (Grone et al., 2010), gray mullet (Nocillado et al., 2007), cobia (Mohamed et al., 2007), fathead minnow (Filby et al., 2008), Atlantic croaker (Mechaly et al., 2009), Senegalese sole (Mechaly et al., 2011), Atlantic halibut (Mechaly et al., 2010), and European eel (Pasquier et al., 2011) belong to the Kiss-R2 subfamily. Therefore, in teleosts, Kiss2-Kiss-R2 is evolutionarily highly conserved and may be functionally equivalent to mammalian Kiss1-Kiss-R.

The nomenclature for two Kiss-R types has been classified based on phylogenetic analysis (Um et al., 2010). Recent genome synteny-based classification studies have clearly demonstrated high conservation of neighboring genes of four fish Kiss-R types (Lee et al., 2009). The fish Kiss1Ra-containing genome fragments have a large array of common neighboring genes, which include ATP6V0B, RABL5, ODF2L, CYR61, C1QL3, GPT, FUZ, CCDC24, and B4GALT2, while the fish Kiss1Rb-containing genome fragments have conserved PSAT1, ZCCHC6, and ISCA1. Ligand-receptor binding assays have shown that while Kiss1 peptide (Kiss1–10) activates Kiss1Rb (GPR54-2) more efficiently than Kiss2 peptide (Kiss2-10) in zebrafish (Lee et al., 2009) and sea bass (Tena-Sempere et al., 2012), Kiss2–10 performs more efficiently in goldfish (Li et al., 2009). Kiss1Ra (GPR54-1) is activated by Kiss1–10 in goldfish (Li et al., 2009) and activated by Kiss1–10 and Kiss2–10 in zebrafish and sea bass (Lee et al., 2009; Tena-Sempere et al., 2012). Interestingly, human Kiss1–10 was an effective homolog in activating Kiss-R2 in the orange spotted grouper (which ordinarily has only Kiss2/Kiss-R2 pair) (Shi et al., 2010). Therefore, in the following sections, fish Kiss1Ra and Kiss1Rb are designated as Kiss-R2 and Kiss-R1, respectively.

### Distribution

Two Kiss-R genes are highly expressed in various reproductive tissues including the brain, pituitary, and gonads and partially in other peripheral tissues (Nocillado et al., 2007; Biran et al., 2008; Filby et al., 2008; van Aerle et al., 2008; Li et al., 2009; Mechaly et al., 2009; Tena-Sempere et al., 2012) (Table 2). Kiss-R gene expression has been described in specific parts and throughout the teleost brain (Biran et al., 2008; Filby et al., 2008; Martinez-Chavez et al., 2008; van Aerle et al., 2008; Shahjahan et al., 2010). In the Senegalese sole, the two Kiss-R isoforms (Kiss1r_v1 and Kiss1r_v2) exhibit differential patterns in the brain (Mechaly et al., 2009). In the brain of fathead minnow, *Pimephales promelas*, *kiss1r* (homologous to Kiss-R2) gene expression is largely seen in various brain regions including the olfactory bulbs, the dorsal and ventral telencephalon, the hypothalamic nuclei, the midbrain, and the hindbrain (Filby et al., 2008). In the zebrafish, *in situ* hybridization has shown that most *kiss-r1* (GPR54-2) mRNA is expressed in the habenula (Ogawa et al., 2010, 2012a; Servili et al., 2011), whereas *kiss-r2* (GPR54-1) mRNA is widely expressed in the fish brain, especially in the olfactory bulb, telencephalon, preoptic area, midbrain, hypothalamic nuclei, cerebellum, and spinal cord (Grone et al., 2010; Servili et al., 2011; Ogawa et al., 2012a) (Figure 1B).

**Table 2:** **Kisspeptin receptors in teleosts**.

Type	Species	Original name	Accession number/ensembl ID	Ligand selectivity	Localization		Reference
					Brain	Pituitary	
Kiss-R1a	Coelacanth	Kiss-R1a	ENSLACP00000018620	ND	ND		
Kiss-R1b	Goldfish	GRP54b	ACK77793	Kiss1 > Kiss2 (SRE)	+	+	Li et al. (2009)
	Zebrafish	GPR54-2/Kiss1Rb	EU047918/ENSDARP00000088021	Kiss1 > Kiss2	+	+	Lee et al. (2009), Servili et al. (2011), Ogawa et al. (2012a)
	Medaka	GPR54-1/Kiss1Rb	ENSORLP00000002102	ND	ND		Lee et al. (2009)
	Coelacanth	Kiss-R1b	ENSLACP00000000327	ND	ND		
Kiss-R2a	Goldfish	GPR54a	ACK77792	Kiss2 > Kiss1 (CRE)	+	+	Li et al. (2009)
	Zebrafish	GPR54-1/Kiss1Ra	EU047917/ENSDARP00000011859	Kiss1 = Kiss2	+	−	Lee et al. (2009), Servili et al. (2011), Ogawa et al. (2012a)
	Cod	Kiss1R	ENSGMOP00000011986	ND	ND		
	Coelacanth	Kiss-R2a	ENSLACP00000019382	ND	ND		
	*Fugu*	Kiss1R	ENSTRUP00000035136	ND	ND		
	Stickleback	Kiss-R	ENSGACP00000022743	ND	ND		
	*Tetraodon*	Kiss1R	ENSTNIP00000017204	ND	ND		
	Tilapia	Kiss1R	ENSONIP00000011710	ND	ND		
	Medaka	GPR54-2/Kiss1Ra	ENSORLP00000022191	ND	ND		Lee et al. (2009)
	Platyfish	Kiss1R	ENSXMAP00000017086	ND	ND		
Kiss-R2b	Coelacanth	Kiss-R2b	ENSLACP00000001512	ND	ND		
	Green anole	GPR54-like	XP_003217188	ND	ND		
	Platypus	GPR54b	XP_001507133	ND	ND		

*ND, not determined. SRE, binding response shown with serum response element; CRE, binding response shown with cAMP response element*.

The distinct expression patterns of two Kiss-R types indicate their specific roles in reproductive and non-reproductive functions in fish. The habenula, a conserved structure in the brain of vertebrates has been shown to play important roles in various brain functions and behaviors, which include circadian rhythmicity, feeding, stress, sleep, affective states, and maternal and sexual behaviors (Teitelbaum and Epstein, 1962; Modianos et al., 1974; Corodimas et al., 1992; Matthews-Felton et al., 1995; Klemm, 2004; Zhao and Rusak, 2005). In mammals, the habenula expresses neuropeptides such as substance P (SP) and neuropeptide Y (NPY) (Neckers et al., 1979; Smith et al., 1985). In rats, GnRH-immunoreactive non-neuronal mast cells have been observed in the habenula (Khalil et al., 2003), which have also been noted in GFP-GnRH transgenic rats (Parhar et al., 2005b). Similarly in the habenula of teleosts, there are groups of cells immunoreactive to neuropeptides such as SP and corticotropin-releasing factor,(Villani et al., 1991; Mousa and Mousa, 2006), and expression of *tac2a*/*tac3a* gene (encoding neurokinin B) has been recently identified in the habenula of zebrafish (Biran et al., 2012; Ogawa et al., 2012b). These neuropeptide containing cells in the habenula may co-express Kiss-R to regulate a variety of neuroendocrine functions. As Kiss-R2 are widely expressed in the preoptic-hypothalamic regions in fish, Kiss-R2 could also be expressed in other neuronal populations in addition to GnRH neurons, which could play a key role in the control of reproduction, as has been suggested in mice (Herbison et al., 2010; Hanchate et al., 2012).

In some teleosts, Kiss-R is also expressed in the pituitary (Filby et al., 2008; Martinez-Chavez et al., 2008; Shahjahan et al., 2010) (Table 2). In the pituitary of goldfish, Kiss-R2 (GPR54a) is expressed in the gonadotrophs, somatotrophs, and lactotrophs (Yang et al., 2010), which corresponds with the existence of fiber innervation of Kiss2-immunoreactive fibers in the pituitary of zebrafish (Servili et al., 2011). In mammals, kisspeptin appears to directly stimulate the secretion of LH, growth hormone, and prolactin secretion (Gutiérrez-Pascual et al., 2007; Kadokawa et al., 2008a,b). These results suggest that the hypothalamic kisspeptin-Kiss-R system regulates the reproductive functions at the level of the brain as well as at the level of the pituitary.

### Expression of Kiss-R in GnRH neurons

Our group (Parhar et al., 2004) was the first to report Kiss-R (Kiss-R2) and GnRH co-localization in tilapia using single-cell gene profiling coupled with laser-captured microdissection. This finding has established the concept that kisspeptin directly regulates GnRH neurons. We demonstrated expression of Kiss-R2 mRNA transcripts in all three GnRH neuronal types in tilapia (Parhar et al., 2004), which has also been confirmed in another cichlid by *in situ* hybridization (Grone et al., 2010). A close association between Kiss2 fibers and GnRH neurons have been established by a recent immunohistochemical study in zebrafish (Servili et al., 2011). In the mullet, Kiss-R2 gene expression positively correlates with GnRH2 and GnRH3 (Nocillado et al., 2007). In the early larval and juvenile cobia brain, Kiss-R (homologous to Kiss-R2) expression and all three GnRH mRNAs remains remarkably similar (Mohamed et al., 2007). These results suggest that Kiss-R2 and GnRH have coordinated roles at early pubertal stages in fish. In zebrafish, *kiss1*, *kiss2*, GnRH2, and GnRH3 mRNA levels are increased at the start of the pubertal phase (Kitahashi et al., 2009), indicating its role in controlling the onset of puberty. A very recent study in the *Morone* species has demonstrated that Kiss-R2 is co-localized in GnRH1 neurons in the preoptic area, while Kiss-R1 is expressed in cells attached to GnRH1 fibers, indicating two different GnRH1 regulatory methods (Zmora et al., 2012). These findings also indicate potential relationships between Kiss-Rs and multiple GnRHs, implicating kisspeptin-Kiss-R in the development and maturation of the piscine reproductive system.

### Kiss-R signaling in teleosts

The ligand specificity of the piscine Kiss/Kiss-R system has previously been demonstrated by analyzing PKC-MAPK activation in several teleosts species (Biran et al., 2008; Lee et al., 2009; Tena-Sempere et al., 2012) (Figure 2B). All piscine Kiss-Rs tested successfully activated luciferase expression with the help of an SRE promoter, indicating the significance of PKC-MAPKs as a signaling pathway (similar to mammals) (Tena-Sempere et al., 2012). However, variances in kisspeptin length and receptor-ligand combinations have resulted in observable differentiation. Mammalian Kiss-Rs require a core sequence of kisspeptin (Kiss-10) to optimally activate Kiss-R (Kotani et al., 2001). All available fish Kiss2 sequences show a conserved Arg (position 13), indicating a putative mature Kiss2–12 peptide, while Kiss1 sequencing revealed a mature Kiss1–15 peptide due to the presence of a conserved N-terminal Gln (Tena-Sempere et al., 2012), which has further been supported by more active kisspeptin results from pyroglutamylation of the N-terminal Gln (Lee et al., 2009). Only zebrafish and sea bass have served in the testing of longer kisspeptins (Kiss1–15 and Kiss2–12) (Lee et al., 2009; Zmora et al., 2012). In all cases, the longer peptides have activated Kiss-Rs more effectively than their respective shorter peptide form with some exceptions (Lee et al., 2009; Tena-Sempere et al., 2012).

The cAMP/PKA pathway is activated more potently by Kiss-R1 than Kiss-R2 in zebrafish, goldfish, and sea bass (Tena-Sempere et al., 2012). Maximum activation of Kiss-R1 in goldfish is achieved with Kiss2–10 (Li et al., 2009), while it is most potently activated by Kiss1–15 in sea bass (Tena-Sempere et al., 2012). The single-ligand test using Kiss1–10 in zebrafish found that the cAMP/PKA pathway could be activated through Kiss-R1 (but not Kiss-R2) activation (Biran et al., 2008). In the orange spotted grouper, where the Kiss-R2 was solely studied, there was no observable PKA signaling pathway (Shi et al., 2010), which is consistent with previous piscine observations that Kiss-R2s have compromised signaling through this pathway. In some species, GPR54 duplicates have exhibited differentiation in signaling. However, further analysis of fish species is required to specify defined signaling patterns (Tena-Sempere et al., 2012).

### Independent function of two kisspeptin-Kiss-R systems

In several teleost species, the onset of puberty marks a significant increase in Kiss-R mRNA expression (Biran et al., 2008; Filby et al., 2008; Martinez-Chavez et al., 2008). GPR54 is only induced in the mullet brain during the pubertal stages, leading to speculation that it also plays a role in reproductive development (Nocillado et al., 2007). In the brain of the fathead minnow, the highest expression of Kiss-R1 mRNA coincides with higher levels of GnRH gene expression in sexually mature females compared to prepubertal females (Filby et al., 2008). Although kisspeptin is considered to be a potent regulator of reproductive function, the specific function of two kisspeptin-Kiss-R systems has not been well understood due to poor ligand selectivity of two Kiss-R types for Kiss1 and Kiss2 peptides (Um et al., 2010). Physiological studies in some teleosts have suggested that Kiss2 exhibits higher potency in reproductive regulation compared to Kiss1 (Felip et al., 2009; Kitahashi et al., 2009; Li et al., 2009; Akazome et al., 2010; Shahjahan et al., 2010). Conversely, in the medaka, Kiss1 but not Kiss2 neurons in the hypothalamus exhibit estrogen sensitivity (Mitani et al., 2010), which suggests that Kiss1 but not Kiss2 plays a role in central reproductive regulation in the medaka (Oka, 2009). In the *Morone* species, Kiss1 is more potent in inducing LH release, with Kiss2 downregulating GnRH1 and Kiss-R2 gene expression during recrudescence (Zmora et al., 2012). These highly diverse observations suggest that the kisspeptin-Kiss-R pathway plays important roles in piscine reproduction; however, given the diversity of reproduction strategies, environmental niches and the timing of sexual maturation, and inconsistency in experimental approaches, it is difficult to establish a unifying theme (Oakley et al., 2009).

A recent study in zebrafish has managed to demonstrate an independent function of two kisspeptin-Kiss-R systems (Ogawa et al., 2012a). In zebrafish, Kiss1 neurons are solely present in the habenula, whereas Kiss2 neurons are only expressed in the preoptic-hypothalamic nuclei (Kitahashi et al., 2009; Servili et al., 2011). Similarly in zebrafish, Kiss-R1 mRNA expression occurs mainly in the habenula, whereas Kiss-R2 mRNA expression occurs widely around the brain (Kitahashi et al., 2009; Servili et al., 2011). This anatomical correspondence between kisspeptin and Kiss-R types clearly indicates that their ligand-receptor pairs functionally represent the key-keyhole interaction in zebrafish. Therefore, zebrafish is an attractive model system to study independent functions of two kisspeptin-Kiss-R systems in vertebrates. We have recently revealed that the Kiss1 neurons in the habenula that project into the interpeduncular nucleus (IPN) could modulate the serotonergic system through an autocrine mechanism (Ogawa et al., 2012a). However, the precise mechanism that underlies this finding and its role in serotonin regulation remains unknown.

### Evolutionary significance of the kisspeptin-Kiss-R system

**Figure 3:**
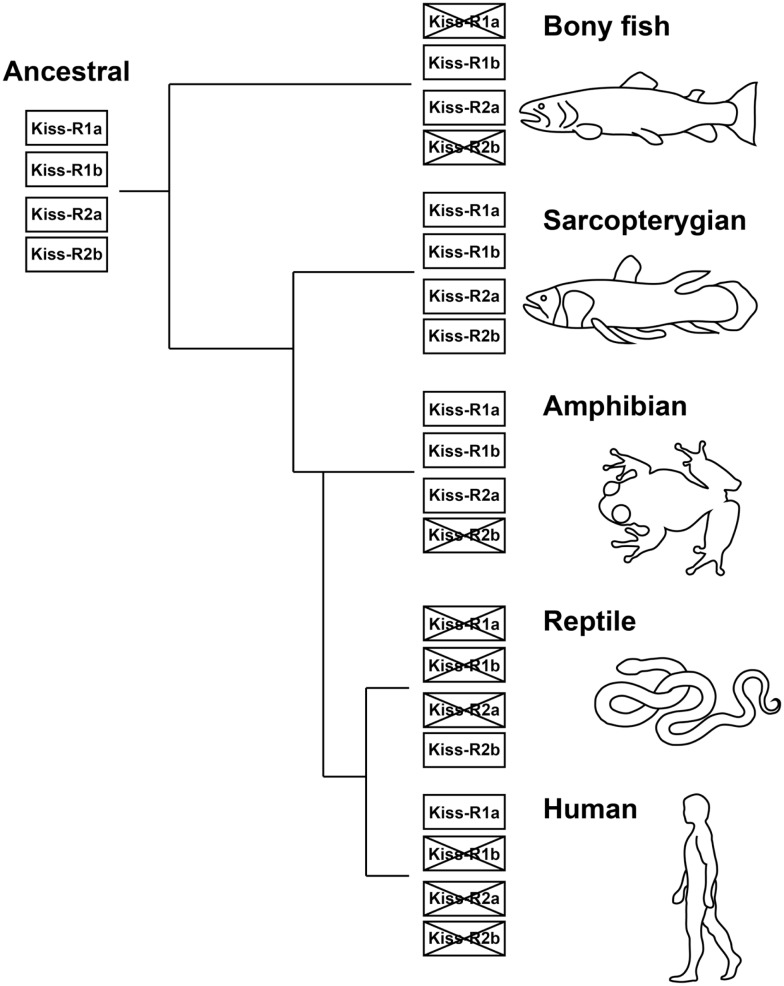
**Proposed molecular evolutionary history of the Kiss-R genes**. Four Kiss-R types originated from two common ancestral Kiss-R genes (Kiss-R1 and Kiss-R2), diversified through gene or chromosome duplication and gene modification and deletion. Four Kiss-R types are still conserved in the Coelacanth genome.

The fishes provide excellent animal models to study the principles that underlie the vertebrate kisspeptin and Kiss-R systems from an evolutionary viewpoint (Akazome et al., 2010). The conserved genomic organization and gene synteny of the *kiss* and *kiss-r* genes indicates that they originated from a common ancestral gene (Akazome et al., 2010; Um et al., 2010). The vertebrate lineage exhibits this conserved genome organization, spanning mammalian, and non-mammalian species (Tena-Sempere et al., 2012). Our genome synteny analysis found four predicted Kiss-R homologous types in the Coelacanth genome (Kiss-R1a, Kiss-R1b, Kiss-R2a, and Kiss-R2b) (Table 2). Genes surrounding Coelacanth Kiss-R1a gene are also found in the genomic region of *Xenopus* GPR54-1a and mammalian Kiss-R genes but absent in fish Kiss-R types. Coelacanth Kiss-R1b is close to fish Kiss1Rb and *Xenopus* GPR54-1b. Neighboring genes of Coelacanth Kiss-R2a were found in a region surrounding *Xenopus* GPR54-2 and fish Kiss1Ra but were absent in the human Kiss-R. Coelacanth Kiss-R2b is close to Platypus GPR54b and Green anole (*Anolis carolinensis*) GPR54-like sequence. Based on the phylogenetic analysis, it can be hypothesized that the two ancestral Kiss-R lineages (Kiss-R1 and Kiss-R2) were first duplicated into four Kiss-R lineages during chromosome duplication in ancestral fish (Figure 3). Among the four lineages, Kiss-R1a and Kiss-R2b lineages have been lost in bony fish, Kiss-R2b lineage could have been lost in amphibian but may exist in reptile and platypus, and only Kiss-R1a lineage is preserved in human. However, in the Coelacanth genome, all four Kiss-R lineages are still conserved. These results correspond with the concept of molecular evolutional history of vertebrate Kiss-R genes that has been previously proposed (Lee et al., 2009; Zohar et al., 2010).

The alternating actions and importance of both Kiss1 and Kiss2 were very recently demonstrated in the *Morone* species (Zmora et al., 2012). They concluded that the organization of the kisspeptin system suggests a transitional evolutionary state between early to late evolving vertebrates. The evolutionary transition between multiple forms of kisspeptin, present in evolutionarily older vertebrates such as frogs and some fish, to a single form, as evident in higher vertebrates, is exemplified in the kisspeptin systems of the various fish species studied thus far (Um et al., 2010; Zmora et al., 2012). In the Coelacanth genome, only one kisspeptin-homologous sequence (ENSLACP00000010201, FNFNPFGLRF) was identified, which is close to fish Kiss2. Genes surrounding the Coelacanth Kiss1 (LDHB, GYS2, SLC25A3, STRAP, and GOLT1B) were found in the Stickleback Kiss2, *Xenopus* Kiss2, and Zebrafish Kiss2, but not in the human Kiss1. Therefore, in the Coelacanth, Kiss1 lineage could have been lost, although genes rounding the human Kiss1 such as REN, ETNK2, and SNRPE are still conserved in the Coelacanth genome. The complete disappearance of Kiss1 and its functional relocation in different fish species clearly shows its evolutionary transition (Zmora et al., 2012). However, the physiological significance of two or loss of one Kiss system in fish species still remains unknown. The evolution of kisspeptin-Kiss-R system may be closely related to the evolution of reproductive traits. It is hence crucial to examine kisspeptin-Kiss-R system in fish species with one-Kiss and those with two-Kiss systems from the viewpoint of diversity of reproductive physiology, i.e., single- or multiple-spawner, seasonal breeder, social or non-social species, viviparous or non-viviparous fish, lifespan, and sex changing fish.

## Concluding Remarks

The hypothalamic GnRH system has been well studied, and the specific functional roles of the various receptor-ligand pairs have been delineated for both mammalian and non-mammalian vertebrates. However, research on the kisspeptin/GnRH relationship in non-mammalian vertebrates, including fish, is still in its infancy. Studies have so far shown in fish that kisspeptins directly regulate GnRH neurons and GnRH release via interactions with Kiss-Rs. However, there are numerous unknown or unconfirmed (confirmed in mammals but not in fish) matters regarding the GPCRs in fish reproduction. Understanding the precise mechanism of endocrine regulation of fish reproduction based on GPCRs is necessary to determine the precise physiological roles of kisspeptin-GnRH pathways.

## Conflict of Interest Statement

The authors declare that the research was conducted in the absence of any commercial or financial relationships that could be construed as a potential conflict of interest.
